# Visualizing post genomics data-sets on customized pathway maps by ProMeTra – aeration-dependent gene expression and metabolism of Corynebacterium glutamicum as an example

**DOI:** 10.1186/1752-0509-3-82

**Published:** 2009-08-23

**Authors:** Heiko Neuweger, Marcus Persicke, Stefan P Albaum, Thomas Bekel, Michael Dondrup, Andrea T Hüser, Jörn Winnebald, Jessica Schneider, Jörn Kalinowski, Alexander Goesmann

**Affiliations:** 1 Computational Genomics, Center for Biotechnology, Bielefeld University, Bielefeld, Germany; 2 Institute for Genome Research and Systems Biology, Bielefeld University, Bielefeld, Germany; 3 International NRW Graduate School in Bioinformatics and Genome Research, Bielefeld University, Bielefeld, Germany

## Abstract

**Background:**

The rapid progress of post-genomic analyses, such as transcriptomics, proteomics, and metabolomics has resulted in the generation of large amounts of quantitative data covering and connecting the complete cascade from genotype to phenotype for individual organisms. Various benefits can be achieved when these "Omics" data are integrated, such as the identification of unknown gene functions or the elucidation of regulatory networks of whole organisms. In order to be able to obtain deeper insights in the generated datasets, it is of utmost importance to present the data to the researcher in an intuitive, integrated, and knowledge-based environment. Therefore, various visualization paradigms have been established during the last years. The visualization of "Omics" data using metabolic pathway maps is intuitive and has been applied in various software tools. It has become obvious that the application of web-based and user driven software tools has great potential and benefits from the use of open and standardized formats for the description of pathways.

**Results:**

In order to combine datasets from heterogeneous "Omics" sources, we present the web-based ProMeTra system that visualizes and combines datasets from transcriptomics, proteomics, and metabolomics on user defined metabolic pathway maps. Therefore, structured exchange of data with our "Omics" applications Emma 2, Qupe and MeltDB is employed. Enriched SVG images or animations are generated and can be obtained via the user friendly web interface.

To demonstrate the functionality of ProMeTra, we use quantitative data obtained during a fermentation experiment of the L-lysine producing strain *Corynebacterium glutamicum *DM1730. During fermentation, oxygen supply was switched off in order to perturb the system and observe its reaction. At six different time points, transcript abundances, intracellular metabolite pools, as well as extracellular glucose, lactate, and L-lysine levels were determined.

**Conclusion:**

The interpretation and visualization of the results of this complex experiment was facilitated by the ProMeTra software. Both transcriptome and metabolome data were visualized on a metabolic pathway map. Visual inspection of the combined data confirmed existing knowledge but also delivered novel correlations that are of potential biotechnological importance.

## Background

To obtain a complete understanding of the function of cells, it is important to identify the roles of genes and their products. The analysis of gene transcripts (transcriptomics) and proteins (proteomics) is accelerated through the use of microarrays, ultra fast sequencing, and mass spectrometry. Additionally, cells contain numerous other organic molecules not directly encoded in the DNA, the metabolites, which are critical for cell function. Knowledge about metabolites is crucial for an understanding of most cellular phenomena [1-3]. All of these "Omics" technologies are also known as post-genomics.

Integrated approaches combining metabolomics with transcriptomics and proteomics have been reported [4,5] and resulted in more detailed insights than any of these approaches for itself.

### Data Visualization

The aim of scientific data visualization is to display properties of a data set that help researchers to identify quickly its most important characteristics. In functional genomics and post-genomic techniques there are recurring visualization strategies that are generally favored by researchers. For example in metabolomics, a frequent way in which molecular biologists like to visualize data is through the use of metabolic pathway maps. For this purpose a several packages and tools have been implemented which will be presented in the next section in more detail. We will highlight the important features and limitations of existing approaches which led us to the decision to implement the web-based ProMeTra tool which is the focus of our work.

### Existing Systems and Pathway repositories

Initially, there were databases such as KEGG [6] or the different realizations of MetaCyc [7] that store information about the structure of metabolic networks. These databases represent static knowledge of metabolic pathways of organisms from all three domains of life. The contained data have been collected and curated over the years of genomic research and can be presented using images of metabolic pathways linking metabolites and enzymes.

Several tools have been developed to visualize and analyze biological networks together with data obtained from functional genomics measurements. Most interesting in this context are tools that visualize experimental data in the form of biochemical networks. The authors of the VANTED system for advanced data analysis and visualization in the context of biological networks [8] presented a comprehensive review of existing pathway visualization and mapping tools such as Cytoscape [9], MapMan [10], KaPPA-View [11], PathwayExplorer [12], and the Viewer included in MetaCyc-related databases [13] such as AraCyc [14]. They pointed out that often only two conditions can be compared. In experiments designed to provide the basis for simulation in systems biology this is of limited use since often changes in metabolite concentration or transcript levels can only be understood if time series experiments are conducted and analyzed. It is also stressed by the authors that most tools are limited to transcriptomics datasets and only Omics Viewer [15], Cytoscape, and MapMan are designed to also display metabolite or other data. A severe limitation of some of the existing tools is their dependency on static maps, i.e. the data is mapped onto predefined pictures. This might be appropriate if the tools are being developed for a single organism or metabolic pathway but in general it clearly limits the re-usability of the approach. We will present some of the main tools and their important features in more detail.

#### Celldesigner and SBML

SBML, the Systems Biology Markup Language, facilitates the description of models and enables their exchange between various simulation and analysis tools. The XML-based SBML is a free and open format distinguished to represent biochemical reaction networks via a clear notation system [16]. The CellDesigner Software is a process diagram editor for visualization and modeling of biochemical networks and gene-regulation. As SBML-compliant Java software it enables the integration of SBW (Systems Biology Workbench) simulation modules [17]. CellDesigner uses a human-readable diagrammatic representation and proposes a set of notations that enforces the established SBML notation [18]. A metabolic pathway created in CellDesigner is a state transition diagram with complex node structure that represents vertexes, state nodes (SN) and transition nodes (TN) and edges between SN and TN (ST-Edge) or rather TN and SN (TS-Edge). A process diagram (PDN) is defined as PND = (SN, TN, ST-Edge, TS-Edge). Each SN has a graphical symbol, for example a protein or gene symbol. Additionally the nature of a reaction, such as catalysis or inhibition, is represented by a symbol for each type of TN [18]. Whereas the CellDesigner pathways consist of well structured and strictly typed entities, users may not include additional descriptive graphical elements.

#### KEGG Pathways and KEGG Markup Language

A major component of KEGG, the Kyoto Encyclopedia of Genes and Genomes, is the PATHWAY database which represents most of the known metabolic pathways [6]. The database is continuously updated and consists of a collection of graphical diagrams, the so called pathway maps. In these maps, a box represents an enzyme and a circle a metabolic compound. The manually drawn and annotated pathway maps represent knowledge about the metabolism, genetic information processing, and cellular processes.

The KEGG Markup Language (KGML) is an XML-based exchange format and contains computerized information about graphical objects and their relations in the KEGG pathways. In KGML a *pathway *element is the root element that specifies one graph object. The nodes of the graph object are represented by the *entry *elements, whereas the *relation *and *reaction *elements specify the edges . An *entry *element contains information about a node of the pathway, like *id*, *name *and *type*. The *relation *element specifies a relationship between two proteins or protein and compound, which is indicated by an arrow. The *reaction *element describes the chemical reaction between substrates and a products . XML-files, which are defined by the KGML schema, can be downloaded from .

#### KaPPA-View

KaPPA-View is a web-based tool and was developed to represent quantitative data for individual transcripts as well as metabolites on plant metabolic pathway maps. The aim of the system is to support the generation of hypotheses of gene function in the metabolic pathways through an intuitive visualization of the transcripts and metabolites. The system uses SVG vector graphic images for the representation of the biochemical pathways and the experimental datasets that are mapped on the pathway representations [11].

#### MapMan

MapMan is a user-driven tool that displays large datasets (e.g. gene expression data from *Arabidopsis thaliana *Affymetrix arrays) onto diagrams of metabolic pathways or other processes. It has been developed specifically for data generated in *Arabidopsis thaliana *experiments measuring transcript or metabolite levels. The visualizations focus is on the display of experimental data in hierarchical and pre-defined pathway maps.

The functionality of KaPPA-View and MapMan can be accessed via web-applications. In general, a tendency to provide sophisticated analysis methods for functional genomics experiments and datasets through web-based frameworks can be observed. Recent examples are the Babelomics project [19] or the DAVID [20] database and analysis tools focussing on e.g. the functional profiling of genome scale experiments. The advantage of web-based analysis tools compared to stand-alone applications is the ease of updates and the possibility to rapidly release new features. Apart from recent web-browsers no additional software needs to be installed by the user.

To summarize, tools such as KaPPA-View or MapMan focus on a limited set of organisms and user defined pathway maps for other organisms or related strains are not supported. Additionally, the means to visualize metabolic pathway information is usually limited by the underlying pathway model as can be seen in the CellDesigner and KEGG pathways. Informative legends or additional user definable graphical elements that explain details are in general not supported.

Whereas most of the aforementioned tools allow to directly upload files with numerical results from "Omics" experiments in simple text-based files (CSV, TSV) or spreadsheets, the support to directly access "Omics" databases containing experimental results via Web Services is to our knowledge not well established. Data integration using Web Services is an elegant method to connect heterogeneous "Omics" frameworks and we will explore this approach for the following functional genomics experiment which combines transcriptomics and metabolomics measurements.

### Corynebacterium glutamicum and lysine production

The Gram-positive soil bacterium *Corynebacterium glutamicum *is widely used for the production of industrially important amino acids. L-glutamate (1.5 million tons) and L-lysine (850,000 tons) are the major products and the amino acid market is growing at an annual rate of 7% [21,22]. The high importance of L-lysine in animal nutrition led to extensive research and optimization of L-lysine production strains in the last decades. An important step towards optimized L-lysine production was the development of a strain having a feedback-deregulated aspartokinase by selecting for resistance against the L-lysine analogue, S-(2-aminoehtyl)-cysteine [23]. Later, it was found that a single amino acid exchange led to the feedback deregulation [24]. From that time on, rational strain improvement replaced the classical mutational approaches. For this strategy, it is necessary to understand not only single enzyme reactions, but to understand global metabolic regulation. The first step along this path was the sequencing of the whole genome of *C. glutamicum *ATCC 13032 [25]. This allowed for the comparison of the wildtype sequence to gene sequences obtained from classically derived producer strains, the basis for the pioneering study of Ohnishi *et al*. [26]. The authors showed that the introduction of only three genes from a producer strain obtained by chemical mutagenesis, each carrying a single mutation, into the wildtype strain led to a tremendous increase in L-lysine production. In fact, the production yield of the recombinant strain was better than that of the original producer, since the recombinant strain does only carry the beneficial mutations and grows faster than the original strain, therefore producing similar amounts of L-lysine in a shorter fermentation period.

The three mutated genes genes are the already mentioned *lysC*, *pyc*, and *hom*. The mutation in *lysC *results in the expression of a feedback-deregulated aspartokinase. Likewise, the mutated *pyc *encodes a pyruvate carboxylase with increased activity, resulting in an improved supply of oxaloacetate. Finally, the homoserine dehydrogenase derived from the mutated *hom *allel is less active, i.e. a leaky mutation, decreasing flux of the L-lysine precursor aspartate-*β*-semialdehyde into the threonine, isoleucine and methionine biosynthetic pathways.

The complete genome sequence was also essential to develop methods for genome-wide high-throughput analysis techniques like transcriptome analysis with DNA-microarrays [27,28] and proteome analysis by two-dimensional gel electrophoresis coupled with peptide mass fingerprinting [29,30]. The genome sequence also helped in deriving metabolic models that supported metabolomics with HPLC-MS or GC-MS [31,32] and fluxomics, a combination of ^13^C-tracer experiments, isotopomer modeling, and metabolite balancing [33,34]. It is regarded important for process and strain optimization to use data sets on the global physiological state of the cell during the production process not only using one, but several techniques.

The problem with this strategy is not only to analyze a process with all available techniques, but to interpret the data in relation to each other. The tool ProMeTra supports this process by a combined display of gene expression and metabolome data sets on a chosen metabolic or other pathway of biological relevance.

As an application example, we present the combined display of transcript abundance and metabolite pool data obtained from different time points of a batch-fermentation of the L-lysine production strain *C. glutamicum *DM1730. *C. glutamicum *DM1730 has the mutations *pyc*P458S, *hom*V59A, *lysC *T311I, and *Δpck *introduced into a wildtype genetic background [35]. Although a cultivation in a fermenter reduces respectively abolishes many stresses like shifting pH and temperature by stringently controlling these parameters, there are stress parameters that can not be avoided. Among these is low oxygen stress that appears at high cell densities. Here, we introduced this stress on purpose by switching off oxygen supply. Analysis of the time course data with the help of ProMeTra gave new insights into the physiology of *C. glutamicum *under L-lysine fermentation and low oxygen stress conditions.

## Implementation

**Figure 1:**
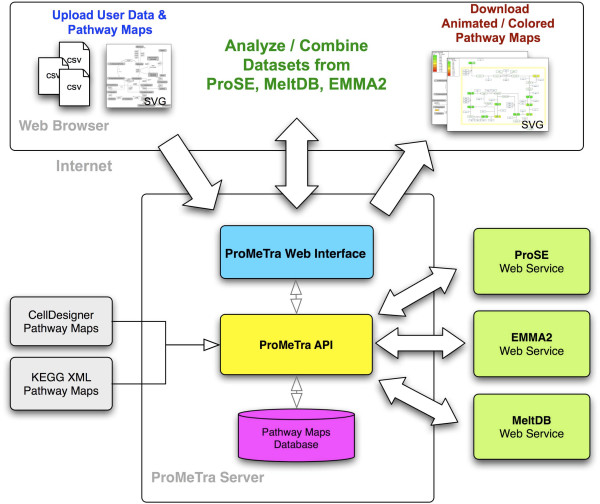
**The ProMeTra system and its data integration concept**. ProMeTra is a web-based system for the integration and visualization of "Omics" datasets. The connection to existing functional genomics platforms such as MeltDB is realized via SOAP-based Web Services. Researchers can upload their own metabolic pathway maps in an annotated SVG format and employ the ProMeTra functionality to render quantitative information originating from transcriptomics, proteomics or metabolomics experiments onto these images. The web interface allows the download of the enriched SVG images. Additionally, methods to upload quantitative results in CSV, or Microsoft EXCEL™ format are provided.

The functionality of ProMeTra can be accessed through a platform independent web application, the general concept of the system is detailed in Figure 1. Perl-based CGI scripts running on an Apache web server dynamically create the HTML-based user interface. The interactivity of the web application is increased through the use of mod perl, JavaScript and AJAX. In order to obtain a flexible and extendable web interface, the Model-View-Controller (MVC) design pattern [36] is employed for the generation of the actual HTML and SVG content. Furthermore, ProMeTra acts as an interactive interface to the experimental datasets stored in the "Omics" platforms MeltDB [37], Emma 2 [38], and Qupe (Albaum *et al*., submitted). Users of these three functional genomic systems can use their existing personalized accounts to employ the data access functionality of ProMeTra and map experimental results. Apart from personalized accounts, a public prometra account is defined. Using the public account, any user can directly log into ProMeTra and upload own datasets and pathway maps. The public ProMeTra account does furthermore provide access to public experiments stored in the previously mentioned "Omics" platforms.

Researchers can access all the preprocessing and visualization functionality of ProMeTra via the web interface. Apart from a recent web browser that supports SVG images directly (e.g. Firefox or Safari) or via specialized plugins (e.g. the Adobe™ SVG viewer for Microsoft Internet Explorer™), no additional software needs to be installed.

Upload of own datasets is possible via CSV formatted files or Microsoft Excel spreadsheets. Details of the supported data formats and the organization of Excel files can be found in the online documentation. The uploaded data files in Excel or CSV format are only stored during a ProMeTra session and are automatically deleted afterwards to ensure the privacy of experimental data. In contrast to the temporarily stored data files, user defined pathway images enriched with information on the presented genes, transcripts, proteins or metabolites can be stored on the ProMeTra server persistently. Every user can decide if his pathways are made public and can also delete and update the uploaded pathway images via the ProMeTra web interface. Information on the pathway maps are stored in an object relational database on the server. User defined SVG pathway maps can be generated using the free Inkscape software available at , the online documentation and the user manual of ProMeTra contain further information on how to install the software and how to generate customized pathway maps.

The core of ProMeTra is an object oriented API that provides access to the pathway maps and the experimental data sets. The main classes are *DataFactory*, *Element *and *Color*. Subclasses of the interface *DataFactory *are responsible for retrieving experimental data from supported data sources. Based on the numerical range of the experimental data, a mapping of various color gradients (e.g. red-yellow-green) is computed by instances of the *Color *class. The functionality to enrich annotated SVG elements in pathway or genome maps is encapsulated in the *Element *class. It provides XML parser functionality to access and extend the DOM tree of any SVG image. The Element class inherits all methods of the XML::DOM::Element class and adds animation and coloring methods. Here, the *Decorator *design pattern was applied in order to attach additional responsibilities to SVG objects and sustain modularity.

### Use of Web Services

We have already shown the successful use of web services to connect heterogeneous software frameworks in functional genomics [39] and also presented the advantages of a tight integration via the BRIDGE layer [40]. The recently established MeltDB, Emma 2, and Qupe systems provide functional genomics datasets through the standardized and interoperable approach of web services. MeltDB and Emma 2 employ SOAP-based web service written in Perl which provide access to normalized quantitative data from metabolomics and transcriptomics experiments. Qupe offers Java-based and WSDL specified methods to obtain the pre-processed experimental datasets originating from quantitative proteomics experiments. ProMeTra is the first web-based system to make use of this functionality and integrates these datasets in one system. For researchers that do not have the possibility to analyze their data using the described web-based systems, we also provide the aforementioned CSV and Excel data import via the ProMeTra web interface.

### Visualization and Animation features

ProMeTra supports SVG images that have been extended by annotations for genes, proteins or metabolites. The images in the open and user readable data format SVG can be uploaded to the web-server via the ProMeTra web interface. We already provide a set of customized pathway images for the industrial amino acid producer *Corynebacterium glutamicum *which is used in the following application example. Metabolic pathways can either be designed and submitted by the user or can be converted via ProMeTra functionality from SBML files defined in CellDesigner. We therefore developed a SBML-to-SVG converter that already includes the mapping of the elements to the KEGG compound database and includes annotated gene locus tags. The mapping of numerical experimental data such as concentrations and ratios is done through a color encoding and rectangles in the SVG image representing genes, proteins or metabolites are subdivided. Therefore the DOM tree of the SVG image is extended by ProMeTra. Child elements are added to the respective rectangles which preserves the original user defined layout.

The number of experimental factors that can be reasonably mapped on a Pathway Map element is limited by its size. For datasets with large numbers of experimental conditions or factors, ProMeTra supports the color animation feature of SVG images. Therefore the background color of an element changes over time which results in an animated SVG image. This feature of SVG images can be visualized in the Opera or Microsoft Internet Explorer web browsers.

ProMeTra offers different color gradients to encode the values of the submitted datasets. Further color gradients can easily be defined with the flexible ProMeTra API. If discrete values instead of M-Value ratios are submitted to the ProMeTra system, the color gradients are computed on the fly ranging from the maximal and minimal values found in the datasets.

It has been pointed out that the representation of "Omics" data on metabolic pathways is most intuitive to the researcher but we also address other concepts of visualization in ProMeTra. We have therefore developed functionality that transforms annotated bacterial genomes present at the NCBI genome repository into so called GenomeMaps. GenBank [41] files of the available replicons are parsed using BioPerl and SVG images (the GenomeMaps) are generated automatically. A GenomeMap represents each annotated coding sequence of a replicon as rectangle in a grid. The order of the rectangles is determined by the chromosomal position of the stop codon of the respective coding sequence and the rectangles are labeled by the associated locus tag or the gene name if present. The grid is filled row after row starting at the top left position for the first gene after the origin of replication. GenomeMaps have been generated for more than 400 bacterial genomes and are available through the ProMeTra web application. An example of a GenomeMap of the chromosome of *C. glutamicum *will be presented in the following application example.

## Results and Discussion

### Fermentation of the strain *C. glutamicum *DM1730 under different aeration conditions

#### Fermentation parameters

**Figure 2:**
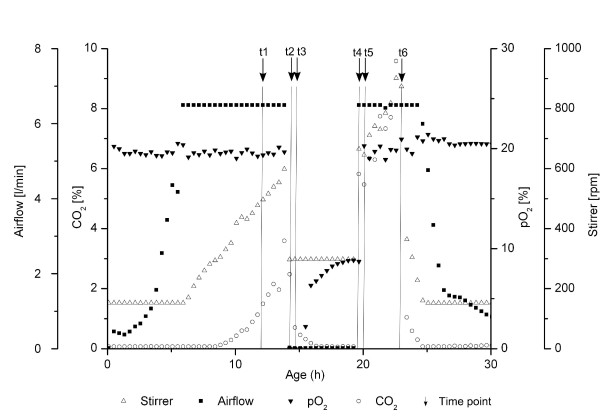
**Online variables of the batch-fermentation of C. glutamicum DM1730**. Online variables of the batch-fermentation of *C. glutamicum *DM1730. Dissolved oxygen (20%) was regulated by a cascade of air flow and stirrer. The black arrows show the harvesting time points at exponential growth (t1), aeration switched off and dissolved oxygen = 0% (t2), 5 minutes dissolved oxygen = 0% (t3), aeration switched on (t4), aeration switched on for five minutes (t5) and carbon source was consumed (t6).

A batch-fermentation of the L-lysine-producing strain *C. glutamicum *DM1730 which was derived from *C. glutamicum *ATCC 13032 [35] was performed under different aeration conditions. Fermentation was in liquid medium with 2.5% glucose as sole carbon source [42], please see Additional file 1 for an in-depth description of the material and methods. The online variables of the fermentation process are shown in Figure 2. First, the culture was grown with 20% dissolved oxygen. The amount of dissolved oxygen was regulated by a cascade of air flow and stirrer. In the middle of the logarithmic growth phase (≈ OD_600 _25), aeration was switched off for about five hours. Afterwards, the aeration was switched on and the culture was grown with 20% dissolved oxygen until the carbon source was depleted. Samples were harvested at six different time points during the fermentation process. The first one (t1) in the logarithmic growth phase after 14.0 hours, the second one (t2) directly after switching off aeration (dissolved oxygen = 0%) and the third one (t3) five minutes after aeration was switched off. During five hours with no aeration the culture apparently stopped oxygen uptake, but a rising amount of dissolved oxygen, most probably originating from air in the fermenter's headspace, was determined in this phase. The fourth sample was harvested directly after switching the aeration on again (t4, 19.6 hours), followed by time point 5 (t5) 5 minutes later. The sixth sample (t6, 23.0 hours) was taken when the carbon source glucose was consumed, indicated by the rapidly decreasing CO_2 _production.

#### Offline variables of the fermentation

**Figure 3:**
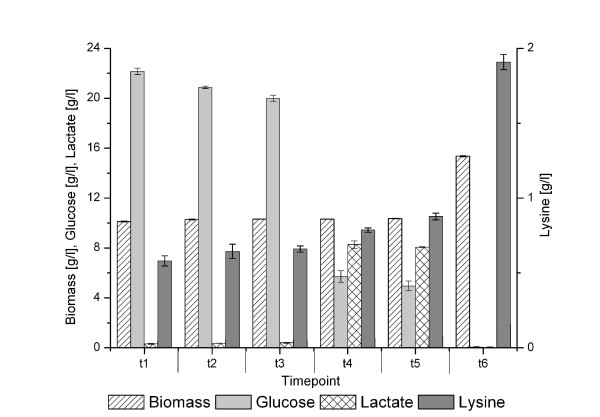
**Offline variables of the fermentation of C. glutamicum DM1730**. Offline variables of the fermentation of *C. glutamicum *DM1730. The figure shows the concentrations of biomass, glucose, lactate and lysine at the six harvesting time points t1 to t6.

At six different time points of the fermentation (t1-t6), samples were harvested in duplicate. They were used for the determination of the concentration of biomass as well as extracellular glucose, lactate, and lysine (Figure 3). The biomass formation stopped after the aeration was switched off and the cells resumed growth after aeration was started again. Glucose was consumed over the whole fermentation, even when the dissolved oxygen was zero. During this period of the fermentation, most of the lactate was produced (between t3 and t4) as shown earlier by Inui *et al*. [43]. External L-lysine was found at all time points. This has also been observed by Takeno *et al*. [44]. The highest amount of lysine was produced in the period between t5 and t6.

### Visualization of single analysis experiments – transcriptome analysis

**Figure 4:**
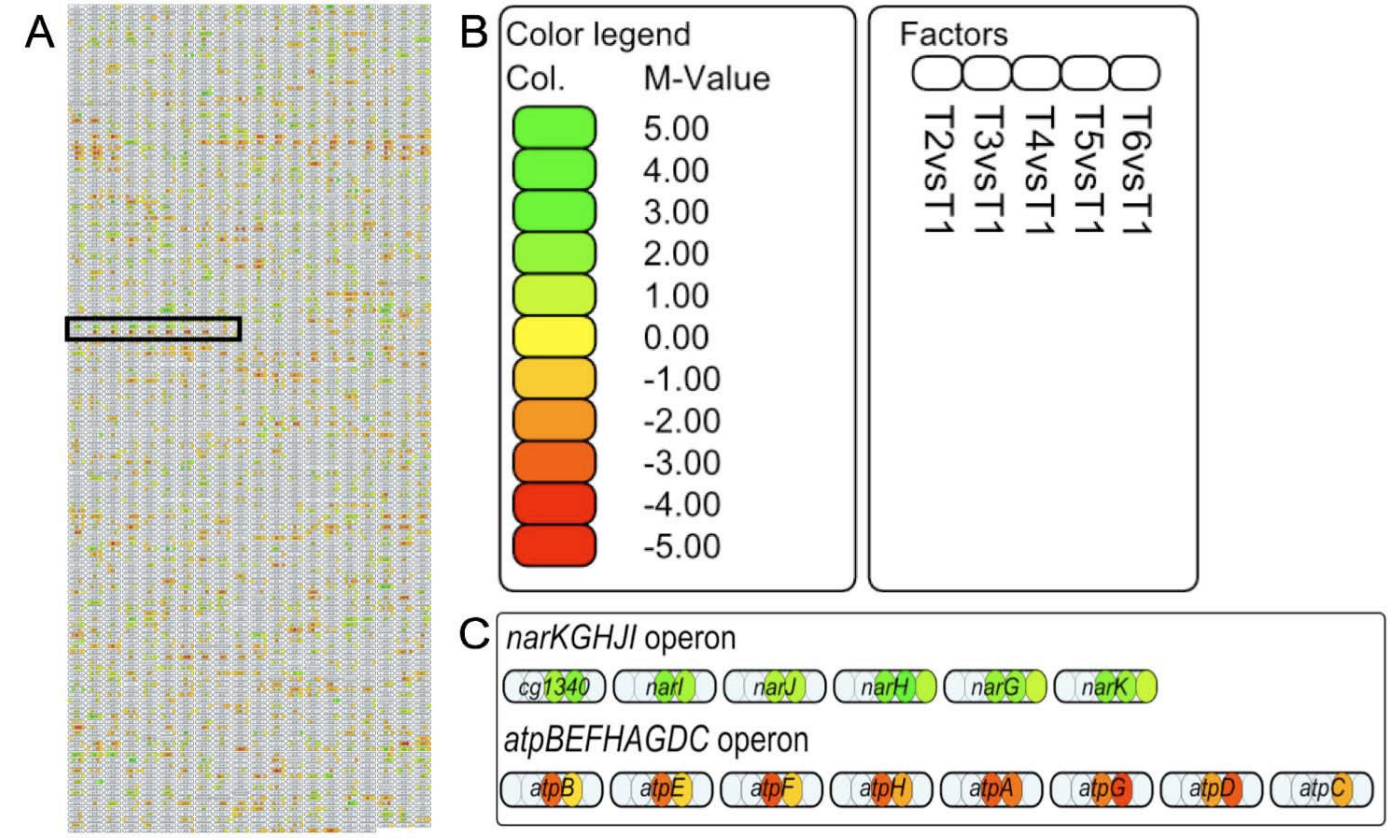
**GenomeMap visualization of the transcriptome analysis**. Visualization of the transcriptome analysis on a genome map. Presented in rounded rectangles are all 3057 genes of *C. glutamicum*, arranged in rows starting with *dnaA *(cg0001) top left, ending with cg3434 down right (A). The presented experimental factors described in the legend of the image (B) are the mean values (M-values) of replicate measurements of the time points T2 to T6 in comparison to the mean values of time point T1. The transcriptome data is represented as five divisions (T2 to T6) of the rectangles. ProMeTra allows to employ different coloring schemes, in this example we have chosen a green to red color mapping for the M-values ranging from 5 to -5. Only those values are displayed that had an error probability less than 5% in a Students *t *test. Part (A) of the figure gives an overview of the transcriptional regulation on the genomic scale. The original SVG image from ProMeTra can be zoomed to any level of detail and we enlarge the marked region of interest in part (C) of the figure. Here, the two transcriptional units of the *nar*-operon (transcribed from right to left) and the *atp*-operon (transcribed from left to right) are presented. For both operons, the genes show a concerted change in regulation at timepoints T4 and T5.

One feature of the software tool ProMeTra is to visualize transcriptome analysis on whole genome maps via color codes. The maps are build from the information available in the NCBI genome database, displaying the genes arranged in rows. Transcriptional units can be identified by the same or similar colors resulting from a similar regulation. Figure 4 shows the visualization of the whole genome map of *C. glutamicum *ATCC 13032 [25] with 3057 annotated genes. As an example for differentially regulated transcription units, the zoomed region of the map displays two regions showing a consistent coloring, the *nar *operon specifying nitrate uptake and nitrate reduction to nitrite and the *atp *operon encoding the subunits of the ATP synthetase.

#### Up regulation of the nar operon under oxygen-limiting conditions

In aerobic bacteria, oxygen is required as exogenous electron acceptor in respiration. The aerobic electron transfer chain in *C. glutamicum *is branched, one branch operates via menaquinone and the other via cytochrome [45]. Under low-oxygen conditions, the anaerobic electron transfer is processed via nitrate respiration by nitrate reductase NarGHJI [44,46]. The genes of the *nar *operon comprise (in the direction of transcription) a putative nitrate/nitrite transporter (NarK), a respiratory nitrate reductase enzyme (NarGHJI) [46], and a transcriptional regulator of the whole operon (ArnR). This regulator acts as transcriptional repressor of the *nar *operon under aerobic conditions [47]. Here, the expression analysis of the *nar *operon revealed an increased transcription at t4 and t5 and a possible co-transcription with *arnR*. It was also proposed that *arnR *is co-transcribed with *narKGHJI *under anaerobic conditions [47], although the gene has its own promoter.

#### Down regulation of the atp operon after oxygen depletion

The eight gene *atp *operon of *C. glutamicum *encodes the subunits of the ATP synthetase that uses ATP to build up a proton gradient and can synthesize ATP by using this gradient [48]. The *atp *operon is less transcribed under low oxygen conditions (t4 and t5), correlating with a lowered energy demand in the absence of growth. This phenomenon was also observed by Inui *et al*. [49] under oxygen deprivation conditions. Since ATPase hydrolyses ATP under non-respiratory conditions, cells save energy by reducing ATPase gene expression.

### Combined metabolite pools and transcript abundances

**Figure 5:**
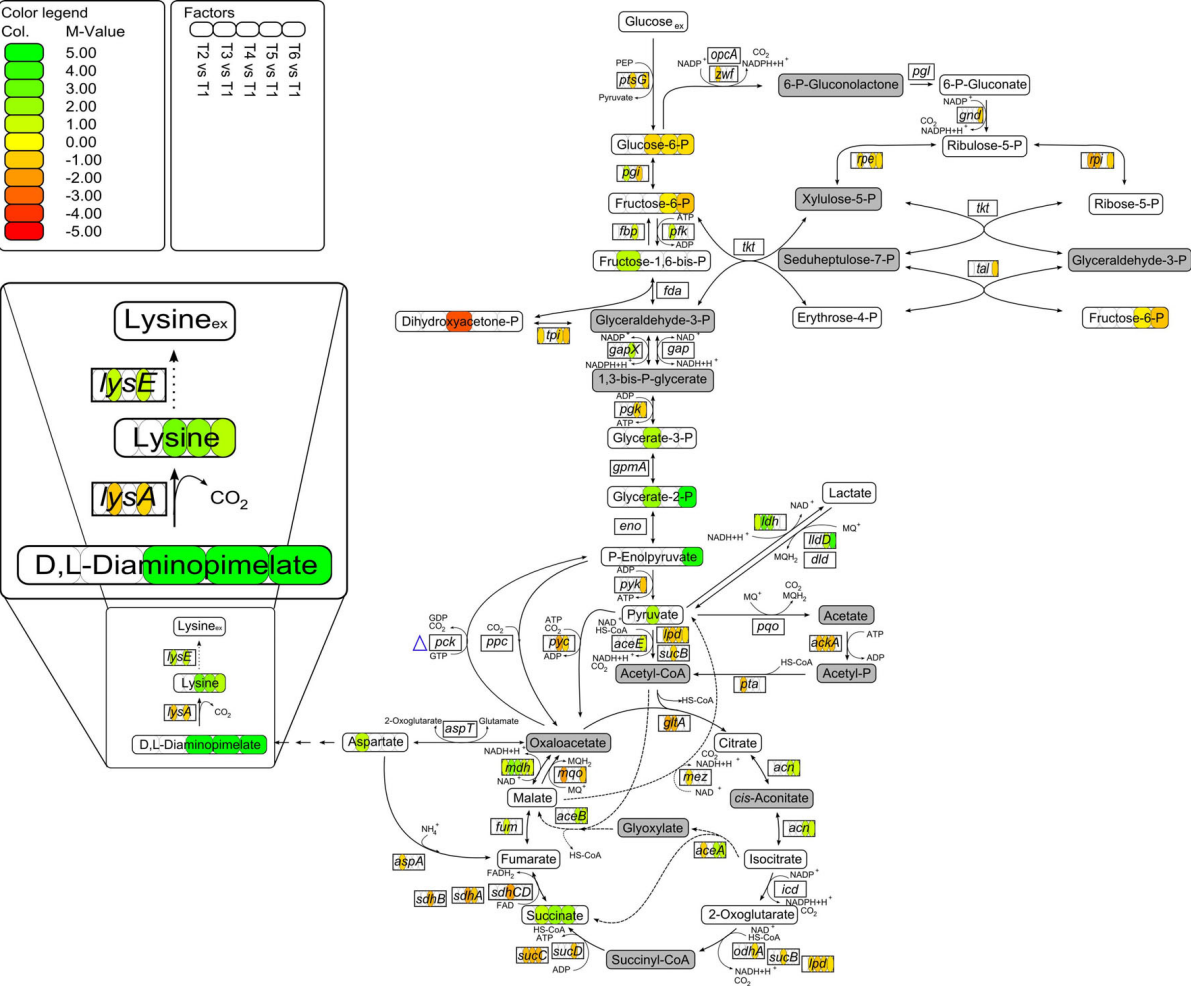
**ProMeTra visualization of relative metabolite pools and transcript abundances during fermentation of the l-lysine producer C. glutamicum DM1730**. ProMeTra visualization of relative metabolite pools and transcript abundances during fermentation of the L-lysine producer *C. glutamicum *DM1730. The metabolite pool ratios are shown in ellipses and the transcript ratios are shown in rectangles as logarithm (base 2) of the mean values (M-values) of time points t2 – t6 in comparison to t1. The values are shown in a color code form green to red with ± 5 as highest values. The pathway map shows glycolysis, pentose phosphate pathway, tricarboxylic acid cycle and the lysine pathway for *C. glutamicum *and a zoomed L-lysine pathway. The pools of the metabolites shown in grey were not determined.

The ProMeTra tool uses the measured values for the relative expression values of the transcripts and the relative pool sizes of the metabolites to map them onto any pathway map (Figure 5). Metabolome analysis was performed by GC-MS using the protocol described by Plassmeier *et al*. [32]. The pathway map shows the main metabolic pathways of *C. glutamicum *from glucose uptake to L-lysine excretion. The L-lysine pathway is shown in a short version, as most of the metabolites between L-aspartate and L-lysine are not identified with GC-MS, because of missing reference substances and non-volatile metabolites. The values of measurements, metabolites, and transcripts are shown in color code from green (upregulated) to red (downregulated). Only those values are displayed that had an error probability less than 5% in a Students t test. For each measurement the value is given relative to that at t1 (logarithmic growth).

Although we were aware of the fact that neither transcript levels precisely predict enzyme activities nor metabolite pools do this for fluxes, a high number of correlations could be identified that correspond with actual knowledge on bacterial metabolism.

#### Lactate consumption and production varies under different oxygen levels

One of the physiological consequence of oxygen depletion for *C. glutamicum *is demonstrated by production and secretion of L-lactate. Lactate production is mediated by the assimilatory lactate dehydrogenase encoded by the *ldh *gene [43]. After aeration is switched off, transcription levels of the *ldh *gene were increasing, coherent with the increasing amount of external L-lactate (Figure 3) [50]. The increasing lactate pool sizes were correlated with increasing internal pool sizes of succinate (t3, t4 and t5). Inui *et al*. postulated that under oxygen deprivation conditions the oxidative arm of the tricarboxylic acid cycle (TCA) is downregulated (*gltA, sucB*, and *sucCD*) and *mdh *(malate dehydrogenase) is upregulated, resulting in a high succinate pool. Downregulation of *gltA *(citrate synthase) leads to an accumulation of pyruvate, which is converted to lactate via lactate dehydrogenase (*ldh*) [49]. Both enzymes, Mdh and Ldh, may regenerate NAD+ to compensate both, downregulation of the oxidative arm of the TCA and the loss of energy-regenerating respiration.

After aeration is switched on again, transcript levels of the *ldh *gene decreased and at the same time (t5 and t6), those of the gene encoding the dissimilatory lactate dehydrogenase *lldA *[51] were increasing. This is consistent with the consumption of external lactate that is metabolized by LldA at the end of the fermentation (Figure 3). Lactate is co-utilized with glucose, a capability of *C. glutamicum *already reported [52]. The observed correlation between lactate utilization and upregulation of the glyoxylate pathway remains unclear. In *C. glutamicum*, carbon sources which enter the metabolism downstream of pyruvate use the glyoxylate pathway as anaplerotic reaction [53]. This is true for single substrate utilization as well as for co-utilization. A reason for the correlation might be that the TCA has to be refilled after downregulation of the oxidative arm of the TCA.

#### Effects on carbon metabolism

In carbon metabolism, drastic differences relative to t1 appeared when all carbon sources had been consumed (t6). Again, several correlations between transcript level and metabolite pool ratios were observed. After glucose was consumed (t6), the transcript level of the gene encoding the glucose-specific enzyme II of the phospho*enol*pyruvate (PEP) phosphotransferase system *ptsG *was lower due to the fact that its transcriptional repressor SugR [54,55] was upregulated (data not shown). Under these conditions cells normally operate gluconeogenesis, which is not possible in DM1730, because the gene for the gluconeogenetic enzyme pyruvate carboxykinase (Pck) was deleted. Under glucose depletion, the gene encoding pyruvate kinase (*pyk*) was found downregulated (t6), possibly to avoid efflux of PEP into the TCA. Probably, due to the fact that at the same time the genes of the two PEP-consuming enzymes PtsG and Pyk displayed lower transcript levels, PEP itself accumulated.

#### Observed variations in production rate of L-lysine

It was apparent that the L-lysine precursor D, L-diaminopimelate accumulated in the cell and the internal L-lysine pool rose after aeration was switched off for a longer time. Diaminopimelate is not only the precursor for L-lysine but also for peptidoglycan biosynthesis, which is used in cell wall production. Since biomass formation and apparently cell wall synthesis almost stopped during low-oxygen conditions, diaminopimelate accumulated (t3, t4 and t5) due to decreased consumption. These higher internal pools stayed almost constant, even after aeration is switched on again and were reflected by higher extracellular L-lysine concentrations (Figure 2). The transcript levels of *lysA *(diaminopimelate decarboxylase) and *lysE *(L-lysine exporter) varied over the fermentation process in an inverse manner. The L-lysine exporter LysE is transcriptionally regulated by LysG [56]. L-lysine is the positive effector of LysG, acting as a sensor for internal L-lysine concentrations. Here, *lysE *was found upregulated at t5, correlating with a high internal L-lysine pool. It was interesting to note that the *lysA *gene encoding diaminopimelate carboxylase, the final step in lysine synthesis, showed an inverse expression behavior. The reason for this is unclear since no transcriptional regulation of the *argS-lysA *operon [57] is known to date.

## Conclusion

We have created the web-based ProMeTra application that is able to visualize and combine data from complex functional genomics experiments. The user-friendly web interface allows researchers to easily visualize and integrate their datasets on pathway maps using the established SVG graphics format. Additional information and graphical content can be added to the vector-based images using drawing software (i.e. CorelDraw™, Inkscape). Unlike other tools such as Omics-Viewer [15] or MapMan [10], ProMeTra is designed to support user designed and annotated pathway maps. This is useful as metabolic pathways which are e.g. available in the KEGG database do not exactly represent the genetic content of the organism under study or genetically modified organisms are analyzed. Furthermore, experimental data stored in functional genomics applications such as MeltDB, Emma 2 or Qupe can be accessed directly via web services. Besides, ProMeTra supports simple CSV and Microsoft Excel files as data input formats. In contrast to commercially available packages such as MetaCore™  by GeneGo and Ingenuity Pathways™  which also provide sophisticated metabolic pathway maps and functionality to map experimental data, ProMeTra uses open standards such as SBML, SVG and Web Services, provides free access to the functionality and offers several public pathway maps. Similar to ProMeTra, the commercially available systems contain a set of predefined pathway maps and allow users to generate their own pathway maps. They also allow to visualize quantitative experimental data from e.g. metabolomics or transcriptomics measurements. Nonetheless, the MetaCore and Ingenuity Pathways system can only visualize results of multiple experiments, time points and dosages through animated graphics according to the systems manuals. This is a limitation since animated pathway visualizations can not be used in publications or on posters. A comprehensive overview of e.g. the progress of a fermentation experiment as presented in Figure 5 can therefore not be generated using the two commercial systems.

The API of the ProMeTra system has an object oriented, modular design. The popular Design Patterns have been used in the creation of the application and allow to easily extend the system to include new data sources and visualization methods. With the use of the MVC approach, we have created a versatile and extendable web interface.

Through the conversion of CellDesigners SBML pathways, we provide means to use the pathway mapping functionality with existing pathway maps and we plan to extend this by conversion of the pathway maps from the KEGG database to our enriched SVG format. In summary, ProMeTra offers a flexible and extendible approach for the analysis, visualization, and integration of functional genomics datasets. Since ProMeTra can access complex experiments processed in MeltDB, Emma 2, and Qupe, we have a system that allows the experienced researcher to focus on the interpretation of the experimental results in an intuitive and visual way rather than on the time consuming conversion of vast tabular data into hopefully meaningful results.

The visualization of transcript abundances from the application example on ProMeTra GenomeMaps confirmed known transcription units and provides an intuitive genome wide overview on transcriptomics datasets. The function of ProMeTra to visualize transcript abundances and metabolite pool deviations onto user-defined pathway maps verified that coherence between transcript level and metabolite pool sizes exist. The visualizations of the application example confirmed existing knowledge and spurred new insights in gene regulation and the corresponding phenotype of cells. In this application example, we used data from two functional genomics techniques, namely transcriptomics and metabolomics. ProMeTra is by design not limited to these data-sources. The pathway map that we employed for this study contained identifiers for the compounds and the transcripts. An extended version of this pathway map that also contains identifiers for the proteins of *C. glutamicum *could easily be generated if additional experimental data becomes available.

## Availability and requirements

ProMeTra is publicly available at . The project info page at  provides further information. We have set up a wiki page together with a user manual in PDF format which details the work flow of a typical ProMeTra analysis, both is available at . Researchers can access the system using the public *prometra *account without further registration. To review the generated SVG images, a recent web browser which supports the SVG image format is needed. For Microsoft Internet Explorer, a SVG plugin is required wich is freely available from Adobe.

## Authors' contributions

The authors would like to point out that this is a joined work of MP and HN and that both have equally contributed to the ProMeTra system and pathway maps. HN has designed and implemented the ProMeTra web application and database. MP has performed the experiments and contributed the biological background and experimental description as well as the biological results and discussion sections. MP has also written the ProMeTra user manual available via the project wiki. TB has implemented an initial prototype and contributed to the development of ProMeTra. JW has contributed to the core ProMeTra API, JS has provided code for the conversion of CellDesigner models. SA and MD have contributed the Qupe and Emma 2 web services and provided substantial intellectual input. AG and JK have supervised the project and manuscript. All authors have read and approved the manuscript.

## Supplementary Material

Additional file 1 **Biological material and methods**. Details on the bacterial strain, media and growth conditions as well as the metabolomics and transcriptomics analyses applied in this study. Click here for file
