# Supplementary material for: Visualizing post genomics data-sets on customized pathway maps by ProMeTra – aeration-dependent gene expression and metabolism of Corynebacterium glutamicum as an example
Source: BMC Syst Biol. 2009 Aug 23;3:82. doi: 10.1186/1752-0509-3-82 (PMC2744654; doi:10.1186/1752-0509-3-82)
Supplement: Additional file 1 — Biological material and methods. Details on the bacterial strain, media and growth conditions as well as the metabolomics and transcriptomics analyses applied in this study. [file 1752-0509-3-82-S1.pdf]

Additional file 1 - Biological material and methods

Bacterial strain

The bacterial strain used in this study was the lysine production strain *C. glutamicum* DM1730 (*pyc*P458S, *hom*V59A, *lysC*T311I, $\triangle$*pck*). The strain DM1730 was derived from *C. glutamicum* ATCC 13032 [1].

Media and growth conditions

For precultures *C. glutamicum* cells were grown at 30°C in CGXII minimal medium [2] containing 30 mgl^{-1} protocatechuic acid. The fermentation was obtained in a 5 L bioreactor (Biostat B, Sartorius AG, Göttingen, Germany) containing $25\,g \cdot l^{-1}$ glucose, $25\,g \cdot l^{-1}$ $NH_4(SO_4)_2$, $1\,g \cdot l^{-1}$ KH_2PO_4, 0,25 $g \cdot l^{-1}$ $MgSO_4$, $10\,mg \cdot l^{-1}$ $FeSO_4$ x $7H_2O$, $10\,mg \cdot l^{-1}$ $MnSO_4$ x H_2O, $1\,mg \cdot l^{-1}$ ZnSO4 x $7H_2O$, 0,2 $mg \cdot l^{-1}$ $CuSO_4$ x $5H_2O$, 0,02 $mg \cdot l^{-1}$ $NiCl_2$ x $6H_2O$, $10\,mg \cdot l^{-1}$ $CaCl_2$ x $2H_2O$, $10\,mg \cdot l^{-1}$ D(+)biotin 2%, $30\,mg \cdot l^{-1}$ protocatechuic acid. The cultivation parameters were pH 7 and 30°C. At the beginning, aeration was adjusted to 20% dissolved oxygen. At an OD_{600} of 25 the aeration was stopped for five hours, then switched on again and operated at 20% dissolved oxygen until the end of fermentation. Diagrams of online variables were created by using the Origin software (Origin Lab, Northampton, USA).

Harvesting time points

Cells were harvested at six different time points. The first sample was taken at middle logarithmic growth phase ($OD_{600} = 25$), the second after aeration was turned off and dissolved oxygen was zero. The third sample was taken at five min growth without aeration and the fourth after 5 hours without aeration. The fifth sample was taken after switching on aeration and 20% dissolved oxygen was reached. The last sample was harvested when glucose was depleted. Each sample was used for the determination of biomass, glucose, lactate, extracellular amino acids, intracellular metabolite pools and transcript abundance analysis.

Biomass, glucose, lactate and amino acid analysis

The optical densities of the cultures were measured at 600nm. The measurement of the biomass concentration (cell dry weight) was performed with the pellet of a 1.5 ml sample of the fermentation broth dried at 37°C. The correlation between optical density and CDW was as follows: CDW = 0.293 x OD (grams per liter).

Extracellular glucose and lactate was measured in duplicate by an enzymatic test system (SuperGL Ambulance, Ruhrtal Labor Technik, Möhnesee, Germany). Therefore, 20 µl of cell free medium is mixed with 980 µl hemolytic solution. A biosensor contains the enzymatic test system, which directly measures glucose and lactate in each probe.

Amino acid analysis was performed by OPA-precolumn derivatization of the cell free media. Separation and quantification of amino acids was done by HPLC (Knauer, Berlin, Germany). For derivatization, the 200 µl media was first mixed with 800 µl methanol. To 250 µl of this solution were added 100 µl borate buffer (0.5 M, pH 8.5), 50 µl OPA-thiol-solution (1% OPA (m/v), 90.5% methanol (v/v), 9.5% borate buffer (0.5 M, pH 8.5), 1% 2-mercaptoethanol), 20 µl HCl (0.75 N) were added. After adding the OPA-thiol-solution, a 1 minute incubation step was performed. 20 µl of the derivatized amino acid solution was injected into the HPLC. As mobile phase, acetate-methanol-solutions were used (buffer A: 50 mM acetate (pH 7.1), 19% methanol; buffer B: 50 mM acetate (pH 7.1), 75% methanol). A gradient of these solutions starting with 100% buffer A for 25 minutes and then rising to 100% buffer B in 60 minutes was used to separate the amino acids on a 300 mm x 3.9 mm *Nova Pak* C18 4 µm column (Waters, Eschborn, Germany).

Metabolome analysis

Metabolome analysis was performed by GC-MS using the protocol described by Plassmeier *et al.* [3]. At each time point of the fermentation duplicate two milliliters culture samples of were taken. Each sample was

treated by short centrifugation, pellet freezing and lyophilisation. After derivatisation by methoxymation and silylation, the measurement of the metabolites was performed in a GC-MS system consisting of a TraceGC gas chromatograph, a PolarisQ ion trap and an AS1000 autosampler (Thermo Finnigan, Dreieich, Germany). For separation of the metabolites, a 30 m x 0.25 mm x 0.25 µm FaktorFour VF-5ms column was used (Varian, Palo Alto, USA). Therefore, 1 µl samples were injected splitless in the 250°C injector. Further parameters were: transfer line temperature 250°C, ion source 200°C, carrier gas flow 1 ml/min. The temperature program was 1 min 80°C followed by rising temperature up to 325°C with a ramp of 5°C/min. The mass spectra were collected with 2 scans/s in a mass range of 50 - 550 m/z. Identification and quantification was performed by the Xcalibur 2.0 software (Thermo Finnigan, Dreieich, Germany) with a database containing the NIST 05 database (NIST, Gaithersburg, MD) and additionally 79 entries generated with purified standards. Metabolite quantities were normalized to the internal standard ribitol and the dry weight of the harvested cells. For data presentation, the mean relative pool size changes of each time point from DM1730 in comparison to time point 1 (logarithmic growth) were calculated. The mean values of those metabolites with an error probability of less than 0.05 in a Student's t-test were regarded significant and used for visualization.

Transcriptome analysis

For transcriptome analysis whole-genome DNA microarrays designed for *C. glutamicum* ATCC 13032 were used. Therefore, 8 µg total RNA from *C. glutamicum* DM1730 fermentation samples were transcribed to cDNA. Labelling of samples and hybridization of the *C. glutamicum* whole-genome DNA microarray were carried out as described previously [4]. DNA microarray hybridization was performed with cDNA from each time point of both strains (Cy5) and the pool of total cDNA of both strains and time points (Cy3). The DNA microarray contains a 70mer oligonucleotide for each *C. glutamicum* gene used as internal technical replicates. Spot detection, image segmentation and signal quantification was performed using the ImaGene 6.0 software (Bio Discovery, El Segundo, USA). Calculation of the intensities of each spot was described previously [5]. For normalization and data evaluation, the EMMA 2.2 microarray data analysis software was used. For data presentation, the mean relative expression changes of each time point in contrast to time point 1 (logarithmic growth) were calculated using a signal intensity ratio (M-value) cut-off of ± 0.6, which corresponds to relative expression level changes equal or greater than 1.5-fold, applying an error probability (P-value) of less than 0.05 according to the Students t test.

References

1. Seibold G, Auchter M, Berens S, Kalinowski J, Eikmanns BJ: **Utilization of soluble starch by a recombinant Corynebacterium glutamicum strain: growth and lysine production.** *J Biotechnol* 2006, **124**(2):381–391, [http://dx.doi.org/10.1016/j.jbiotec.2005.12.027].
2. Keilhauer C, Eggeling L, Sahm H: **Isoleucine synthesis in Corynebacterium glutamicum: molecular analysis of the ilvB-ilvN-ilvC operon.** *J Bacteriol* 1993, **175**(17):5595–5603.
3. Plassmeier J, Barsch A, Persicke M, Niehaus K, Kalinowski J: **Investigation of central carbon metabolism and the 2-methylcitrate cycle in Corynebacterium glutamicum by metabolic profiling using gas chromatography-mass spectrometry.** *J Biotechnol* 2007, **130**(4):354–363, [http://dx.doi.org/10.1016/j.jbiotec.2007.04.026].
4. Brune I, Becker A, Paarmann D, Albersmeier A, Kalinowski J, Pühler A, Tauch A: **Under the influence of the active deodorant ingredient 4-hydroxy-3-methoxybenzyl alcohol, the skin bacterium Corynebacterium jeikeium moderately responds with differential gene expression.** *J Biotechnol* 2006, **127**:21–33, [http://dx.doi.org/10.1016/j.jbiotec.2006.06.011].
5. Brinkrolf K, Plöger S, Solle S, Brune I, Nentwich SS, Hüser AT, Kalinowski J, Pühler A, Tauch A: **The LacI/GalR family transcriptional regulator UriR negatively controls uridine utilization of Corynebacterium glutamicum by binding to catabolite-responsive element (cre)-like sequences.** *Microbiology* 2008, **154**(Pt 4):1068–1081, [http://dx.doi.org/10.1099/mic.0.2007/014001-0].
